# Nanopatterning optimization of zinc phosphide: hole mobility up to 560 cm^2^/V s with selective area epitaxy

**DOI:** 10.1039/d5tc03582a

**Published:** 2026-04-01

**Authors:** Raphael Lemerle, Helena R. Freitas, Thomas Hagger, Didem Dede, Leo Evan Webb, Cynthia Aigroz, Valerio Piazza, Maria Chiara Spadaro, Jordi Arbiol, Anna Fontcuberta i Morral

**Affiliations:** a Laboratory of Semiconductor Materials, Institute of Materials, School of Engineering, Ecole Polytechnique Fédérale de Lausanne 1015 Lausanne Switzerland raphael.lemerle@epfl.ch; b Catalan Institute of Nanoscience and Nanotechnology (ICN2), CSIC and BIST, Campus UAB, Bellaterra Barcelona Catalonia 08193 Spain; c Dipartimento di Fisica e Astronomia “Ettore Majorana”, Universitá di Catania via S. Sofia 64 Catania 95123 Italy; d IMM-CNR, Sede Catania Universitá Via S. Sofia 64 95123 Catania Italy; e ICREA Pg. Lluís Companys 23 08010 Barcelona Catalonia Spain; f Institute of Physics, School of Basic Sciences, Ecole Polytechnique Fédérale de Lausanne 1015 Lausanne Switzerland

## Abstract

Zinc phosphide (Zn_3_P_2_) has been identified as a promising material for low-cost photovoltaics made of earth-abundant elements. In the 90's, the low performance of the devices based on Zn_3_P_2_ was attributed to the poor quality of the crystal. Recently, selective area epitaxy (SAE) has emerged as a method to obtain high quality materials on lattice mismatched substrates, thanks to efficient strain relaxation. Here, we study the relation between the SAE mask characteristics and the resulting optoelectronic properties of Zn_3_P_2_ thin films. Photoluminescence spectroscopy and temperature-dependent Hall measurements outline the impact of those defects on the optoelectronic properties of the thin films. We correlate the effect of the SAE mask pattern on the formation of specific defects such as misfit dislocations and rotated domains. Our results suggest that the rotated domains contribute to the carrier transport by providing p-type carriers while the misfit dislocations are mainly acting as non-radiative recombination centers. Our thickness dependent measurements highlight the key role of the SiO_2_/Zn_3_P_2_ interface, which could induce a highly p-doped region. Overall, these findings drove the optimization of the SAE pattern leading to the highest hole mobility (560 cm^2^/V s) ever measured for this material.

## Abstract 2

This study shows how selective area epitaxy can be used to improve carrier transport in Zn_3_P_2_ and how structural defects, such as rotated domains (RD) interfaces or misfit dislocations (MDs), can affect the optoelectronic properties.

## Graphical abstract

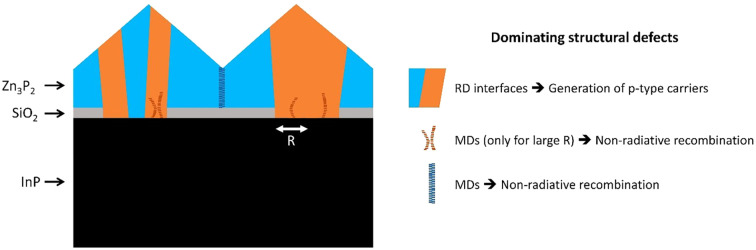

## Introduction

Photovoltaics (PV) is expected to play a crucial role in the energy transition. According to the International Renewable Energy Agency (IRENA), in a scenario of 90% reduction of CO_2_ emissions between 2018 and 2050, the worldwide installed capacity of PV panels should reach 8.5 TW (25% of the global energy generation).^1^ Today, the photovoltaic market is dominated by silicon-based solar cells. Nevertheless, research into alternative materials for solar cells aims at reducing the cost of PV in the future. Among them, Zinc Phosphide (Zn_3_P_2_) is an earth-abundant semiconductor material with promising properties for photovoltaics such as a direct band gap of 1.5 eV and a high absorption coefficient in the visible (≥10^4^ cm^−1^).^2–4^ Interestingly, Zn_3_P_2_ shows intrinsically p-type conductivity because of the low formation energy of intrinsic acceptor defects.^3,5,6^ Still today it is debated whether they originate from Zn vacancies or P interstitials.^5,7^ The most recent computational study from Yuan *et al.* favors the hypothesis of holes generated by Zn vacancies.^8^ Furthermore, Zn_3_P_2_ can accommodate variations in stoichiometry without affecting the crystal structure.^4^ Recently, it has been shown that increasing the phosphorus concentration leads to an increase in the hole concentration from 10^13^ cm^−3^ to 10^19^ cm^−3^.^7,9^

So far, the performance of PV devices based on a Zn_3_P_2_ absorber are still limited. Bhushan *et al.* achieved a record efficiency of 6% for a Zn_3_P_2_/Mg Schottky cell in the 1980s, with no major improvement since.^10^ Several factors could explain the low performances, such as a non-optimized device architecture and a poor quality of the crystal, which results in a low carrier diffusion length within the absorber. Single crystalline epitaxial growth of Zn_3_P_2_ is challenging because of the lack of suitable commercial substrate to grow on. This is due to its large tetragonal unit cell and its high thermal expansion coefficient.^11^ In the majority of the reported studies, the Zn_3_P_2_ has a polycrystalline structure. Recently, monocrystalline Zn_3_P_2_ has been successfully grown by molecular beam epitaxy (MBE) using GaAs or InP as a substrate.^12,13^ However, the high lattice mismatch with the substrate induces strain in the layer with consequent formation of defects, impacting significantly the optoelectronic properties of the material. Bosco *et al.* showed that increasing the thickness of a thin film grown on GaAs leads to an increase in dislocation density, causing a decrease in hole mobility.^12^ Reported hole mobility values lie in the range of 10–300 cm^2^/V s at 300 K for Zn_3_P_2_ thin films and peaks at 310 cm^2^/V s for crystal grown on GaAs.^3^ For thin films grown on InP, the highest mobility measured was 125 cm^2^/V s.^9^ Furthermore, at thicknesses greater than 1 µm, cracks began to form.^9^

In a recent study, we demonstrated the growth of a Zn_3_P_2_ thin film with a low defect density by using Selective Area Epitaxy (SAE) on an InP substrate.^14^ The growth starts in the nanoscale openings, enabling Zn_3_P_2_ pyramidal growth. A thin film forms upon subsequent merging of the pyramids. We showed that the nanostructures significantly relax the accumulated strain without causing the formation of dislocations. Furthermore, the thin films have a textured surface morphology, which can enhance optical absorption.^15,16^ The pattern was fabricated using Electron beam lithography, a costly technique, but this concept could easily be extended to more affordable techniques, such as Talbot lithography or Nanoimprint lithography.^17^

In this work, we correlate the geometry of the pattern -*e.g.* pitch and size of holes into the mask- with the microstructure and the optoelectronic properties of Zn_3_P_2_ thin films grown by SAE on InP(100) substrates. The effect of the growth conditions is also briefly investigated. Transmission electron microscopy (TEM) is used to study the crystal quality, identify the structural defects and determine the chemical composition of the films. Furthermore, we characterize the carrier density and mobility through temperature dependent Hall measurement in a Van der Pauw configuration, getting insight into the defects that dominate carrier-transport mechanisms. Finally, steady-state photoluminescence measurements provide further information on the influence of these defects on the recombination processes in the films.

## Experimental

### Material growth

Fe-doped InP (100) was chosen as growth substrate to exploit its semi-insulating properties (resistivity above 5 × 10^6^ Ω cm) during electrical measurements. To fabricate the mask, a thin layer of SiO_2_ (26 nm) was deposited on the substrate using plasma-enhanced chemical vapor deposition (PECVD), and the SAE pattern was defined using e-beam lithography. The holes were etched by reactive ion etching with CHF_3_/SF_6_ chemistry. The experimental details for the SAE pattern fabrication are further detailed in the SI. The pattern consisted of various 200 µm × 200 µm square arrays of nanoholes. Each array was defined by a specific hole radius (ranging from 45 to 120 nm) and hole pitch (ranging from 200 to 500 nm). The patterned substrate was then introduced inside the MBE system. The equipment used was a Veeco GENxplor Molecular Beam Epitaxy system with a Zn valved cracker cell and a GaP valved cell for the production of P_2_. The sample underwent three annealing steps: 2 h at 200 °C in the load-lock, 1 h at 300 °C in the process module and finally 30 min at 580 °C under a flux of P_2_ (≥10^−6^ Torr) in the growth module. These steps are essential for the removal of surface contaminants, with the final step specifically designed to remove the native oxide of InP.^13^ The growth of Zn_3_P_2_ was then carried out by varying durations, P/Zn ratios and manipulator temperatures depending of the sample. At the end, a thin layer (20 nm) of Si_3_N_4_ was deposited by PECVD to protect the thin film against oxidation.^18,19^

### Device fabrication

In order to perform the Hall measurements on the samples, devices based on the Van der Pauw configuration were fabricated. A schematic of the fabrication procedure can be found in the SI. A first lithography step allowed to selectively etch by plasma etching the Si_3_N_4_ layer at the corners of the square. A second lithography step allowed to sputter a 150 nm layer of Au in contact with the Zn_3_P_2_ exposed. These contacts were connected to larger pads sitting on the Si_3_N_4_ protective layer. Prior to the Au deposition, the sample underwent a 30 seconds Ar sputtering step to remove the native oxide and the deposition of a 5 nm Cr layer to ensure a sufficient adhesion of Au. Finally, the gold pads were wire bonded to a Printed Circuit Board –PCB– following the scheme, described in more details in the SI, required to perform the Hall measurements.

### Measurement methods

The morphology of the samples was determined using a scanning electron microscope (Zeiss Merlin FE-SEM). Cross-section images were acquired with an angle of 20°.

X-ray diffraction (XRD) measurements have been acquired in the rocking curve configuration with an Panalytical Empyrean XRD thin film Diffractometer with a Cu (K-*α*) X-ray source of 1.54 Å.

Electron transparent transmission electron microscopy (TEM) samples were prepared using focused ion beam (FIB) processing at a Thermo Fisher FIB Helios 5 UX. High-angle annular dark field (HAADF) scanning TEM (STEM) images and STEM electron energy loss spectroscopy (EELS) compositional maps were acquired in a double corrected Thermo Fisher Spectra 300 microscope operated at 300 kV, equipped with a Gatan Continuum K3 EELS spectrometer with direct electron detection. Geometrical phase analysis (GPA) has been performed on experimental images by using the licenced GPA plug-in available for Gatan Digital Micrograph to study the layer/substrate epitaxy, identify crystal lattice distortions and rotations, and facilitate the detection of structural defects and induced strain.^20–23^

Prior to the Hall measurements, 4-point measurements were performed to verify the ohmicity of the electrical contacts in the range ±1 V. Temperature-dependent Hall measurements were performed using a physical property measurement system (PPMS DynaCool by Quantum Design). The measurements were performed in low vacuum (≤1 Torr). The excitation current was fixed at 0.1 µA, the magnetic field was swept in the range ±5 T and the temperature was swept between 230 K and 300 K. In theory, the PPMS allows measurements down to 2 K, but the resistivity of the samples was too high for temperatures lower than 230 K. The calculation procedure to extract the resistivity, the carrier concentration and the carrier mobility is described in SI. The activation energy *E*_a_ was calculated from the temperature evolution of the hole concentration *p* in the ionization regime:^24,25^1
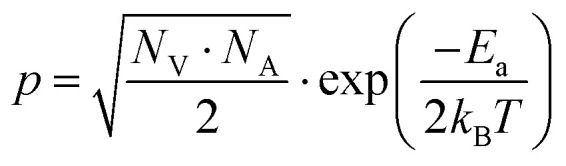
with *N*_V_ the effective density of states in the valence band and *N*_A_ the concentration of acceptor defects.

Finally, photoluminescence (PL) measurements were carried out at room temperature using a 488 nm Ar^+^ laser (25 µA) and a Andor iDus DV420A-OE detector with a laser intensity of 0.42 mW, 1 second of integration time and 5 accumulations. To consider local inhomogeneities, a map of 9 points was acquired and averaged for each sample.

### Samples description

Most of the results presented in this work come from one set of samples: the 270 °C set. This set consists of samples that were grown at 270 °C with a V/II flux ratio of 1.8. We varied the pattern dimensions (pitch and hole radius) and adjusted the growth time accordingly, as discussed later. Hall measurements have been performed on all samples and the temperature evolution is shown in more details for Z1, a sample grown from a 45/400 pattern (45 nm hole radius/400 nm pitch). PL measurements have also been performed on all samples and emphasis is put on Z2, a sample grown from a 120/400 pattern. Furthermore, three samples with different patterns (45/200, 45/400 and 120/400) were selected for TEM analysis.

Additionally, two other sets of samples have been prepared: a 290 °C set and a *T*-variation set. The 290 °C set is similar to the 270 °C set except that the growth temperature was fixed at 290 °C and the flux V/II ratio at 0.9. The *T*-variation set consists of samples grown varying the flux ratio and the temperature. PL measurements have been performed for both sets. Hall measurements have been performed for several samples of the *T*-variation set. Finally, XRD measurements have been performed for the 290 °C set.

## Results and discussion

### Surface morphology

We start by reporting on the evolution of the surface morphology during growth and how the geometry of the pattern influences this evolution. As soon as the nanostructures start to grow out of the openings, they tend to form pyramids with (101) facets.^14^ DFT simulations showed that these facets have the lowest surface energy.^26^ The growth continues until the pyramids coalesce to form a thin film. The lateral growth over the mask is relatively slow. This means that the distance between two openings, affects the overall thickness of the resulting thin film. We observed that for a fixed growth duration, decreasing the pitch and increasing the hole size resulted in a thicker film. Therefore, to obtain thin films of similar thickness, it is important to adapt the growth time to the pattern. The duration of growth in this study ranges from 2.5 hours to 6 hours, resulting in film thickness ranging from 200 nm to 1 µm. More details on the approximations used to determine the film thickness can be found in the SI.

**Fig. 1:**
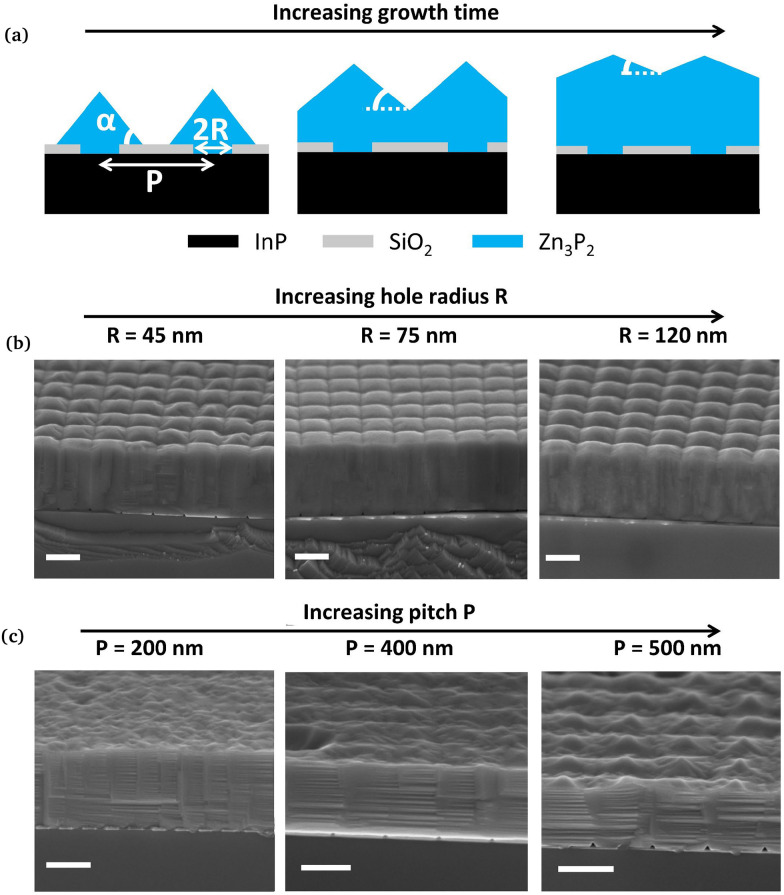
Morphology evolution of the thin films: (a) schematic of the morphology evolution with the thickness, (b) cross-sectional SEM for thin films with various hole size, (c) cross-sectional SEM for thin films with various pitch. All scale bars are 400 nm.

Fig. 1a presents the growth evolution. When fully formed, the pyramids have an angle *α* equal to 55°.^27^ After merging, the angle decreases as the thickness increases. This effect is more or less pronounced, depending on the pitch. For samples of similar thickness, the change in hole size does not influence the surface morphology (see Fig. 1b). On the other side, decreasing the pitch leads to a smaller angle (see Fig. 1c).

### Microstructure of Zn_3_P_2_ thin films

**Fig. 2:**
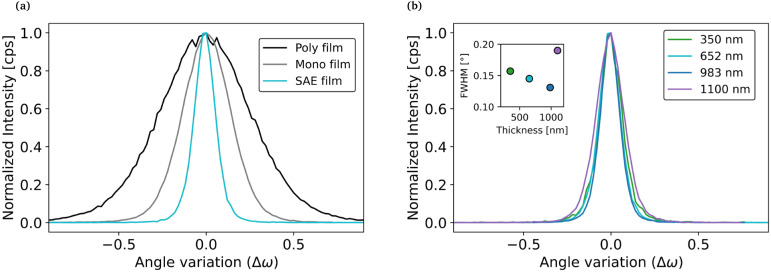
Rocking curve of the Zn_3_P_2_(004) peak with normalized intensity. (a) Comparison between 2D grown (polycrystalline and monocrystalline) and SAE grown thin film and (b) thickness evolution for SAE thin films. The SAE thin films have been grown from a 45/200 pattern at 290 °C.

We now turn towards the study of the microstructure of SAE-grown Zn_3_P_2_ thin films. First, the crystal quality was assessed by X-ray diffraction (XRD) measurements in the rocking curve configuration. The measurements are shown in Fig. 2. The full width at half maximum (FWHM) of the (004) peak, which is representative of the dislocation density,^28,29^ is considerably smaller for thin films grown by SAE compared to films grown directly on InP, whether they are monocrystalline or polycrystalline. Furthermore, we find that the FWHM of SAE films does not increase with the thickness up to 1 µm.

**Fig. 3:**
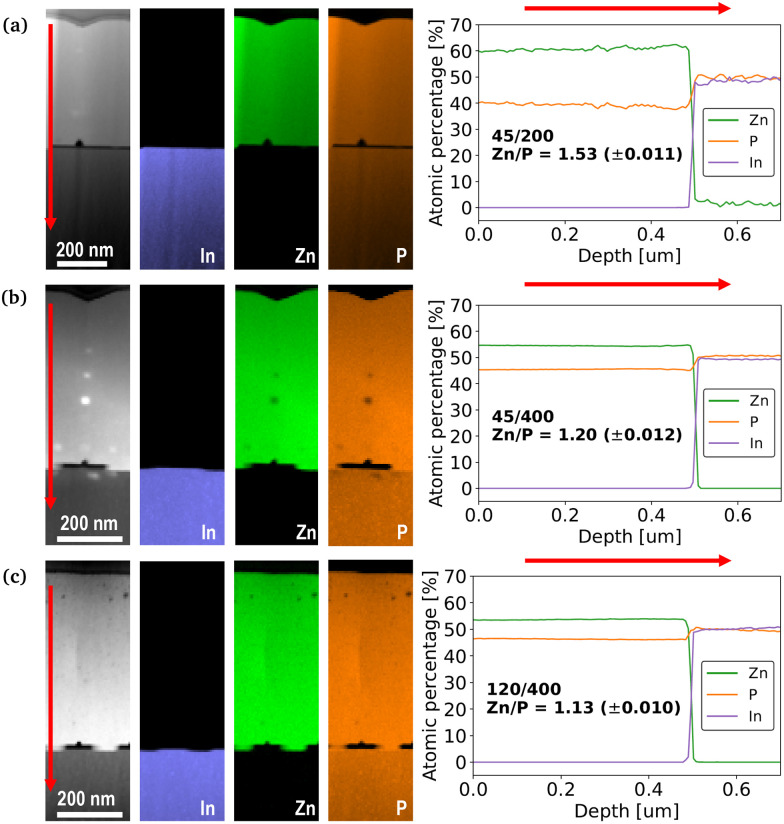
HAADF STEM images and corresponding EELS maps, together with the quantitative compositional linescan along the depth of the sample for (a) 45/200, (b) 45/400 and (c) 120/400 patterns.

The investigation continues with the analysis of the material composition of samples grown with different pattern dimensions using STEM-EELS. The results from EELS linescan quantitative measurements, shown in Fig. 3, provide an overview of the elemental distribution of In, Zn, and P along the depth profiles of the three samples. These data are used to determine the integrated compositional ratios of Zn and P for each sample. Fig. 3a shows a stoichiometric composition of Zn_3.01_P_1.99_ (Zn/P = 1.53) from the sample with the smallest pitch (45/200). As shown in Fig. 3b, increasing the pitch (45/400) leads to an increased phosphorus concentration, resulting in an off-stoichiometric composition of Zn_2.73_P_2.26_ (Zn/P = 1.20). Furthermore, as shown in Fig. 3.c, the sample with the largest hole size (120/400) presents the most phosphorus-rich composition, Zn_2.62_P_2.37_ (Zn/P = 1.13). The reason of this trend is still quite unclear. Furthermore, the trend in hole size contradicts what Escobar *et al.* reported in a previous study.^14^ This suggests that the pattern dependence of the thin film composition may be influenced by the growth conditions. Finally, we note that none of the samples investigated contained any indium from the substrate up to the detection limit and that all thin films presented a homogeneous distribution of Zn and P.

**Fig. 4:**
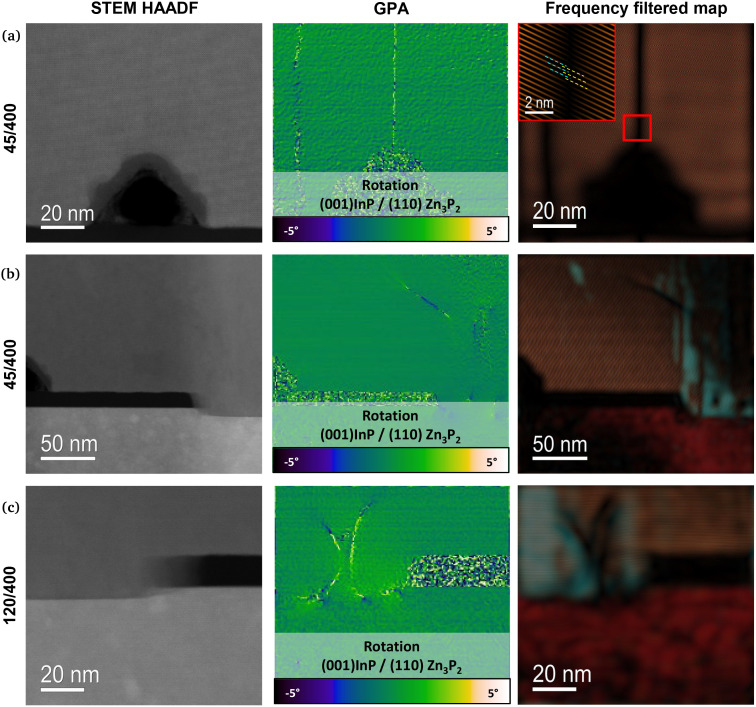
HAADF STEM images for two samples grown at 270 °C. (a) Merging area for the 45/400 sample with the GPA rotation map showcasing the presence of misfit dislocations and the frequency filtered map showcasing no rotated domains, (b) right-side of the mask for the 45/400 sample with the GPA rotation map which does not highlight the presence of misfit dislocations and the frequency filtered map showcasing rotated domains, (c) left-side of the mask for the 120/400 sample with the GPA rotation map highlighting the presence of misfit dislocations and the frequency filtered map showcasing rotated domains.

Finally, to gain a more comprehensive understanding of the microstructure, we focus on identifying defects in the films. This is accomplished using double aberration-corrected HAADF STEM.^30^ The efficiency in acquiring images with an improved signal-to-noise ratio and increased resolution allows us to delve deeper into understanding the modifications of the atomic scale and local changes at the triple interface (InP/SiO_2_/Zn_3_P_2_).^31,32^ In addition, the use of strain mapping allows to precisely measure local displacements, thereby revealing lattice translations, dilatations, and rotations. These approaches provide a comprehensive understanding of the structural properties of the samples investigated.^33,34^Fig. 4 shows images acquired by HAADF STEM measurements for different areas of the film. From the HAADF STEM image of the merging area of a 45/400 sample (Fig. 4a), we detect the presence of few misfit dislocations at the merging interface. Through GPA analysis, we point out to the plane relative rotation for the Zn_3_P_2_ films with respect to the InP substrate which highlight the presence of misfit dislocations along the merging interface. Fig. 4b shows an HAADF STEM image of the InP/SiO_2_/Zn_3_P_2_ triple interface for the same sample. From the frequency filtered map, we can observe the presence of a main domain (Zn_3_P_2_ [100] × (001)), reported in the frequency filtered map in cyan, and a secondary rotated domain embedded in the main one (Zn_3_P_2_ [1̄11] × (110)), here shown in orange. Spadaro *et al.* already detected the multidomain behavior of SAE-grown Zn_3_P_2_ on InP nanowires.^30^ The corresponding GPA rotation map shows a Zn_3_P_2_/InP interface free of misfit dislocations for this sample. Finally, Fig. 4c shows a HAADF STEM image of the InP/SiO_2_/Zn_3_P_2_ triple interface for a 120/400 sample. The presence of rotated domains is also detected from the frequency filtered map. For this sample, the GPA rotation map highlights the presence of misfit dislocations at the Zn_3_P_2_/InP interface. This was only observed for samples with a large hole size, suggesting that these defects could arise from the relaxation of the accumulated strain.

The combination of XRD and HAADF-STEM measurements allows to demonstrate the high quality of the films grown by SAE. Furthermore, EELS measurement reveals an influence of the pattern geometry on composition. By increasing the pitch and the hole size, it results in an increase in the phosphorous content. Finally, structural defects that could affect the optoelectronic properties have been identified in the samples. These defects include misfit dislocations at the merging area and rotated domains. Increasing the hole size leads to the formation of additional misfit dislocations at the Zn_3_P_2_/InP interface while reducing the pitch might increase the occurrence of rotated domains.

### Temperature evolution of electrical properties

**Fig. 5:**
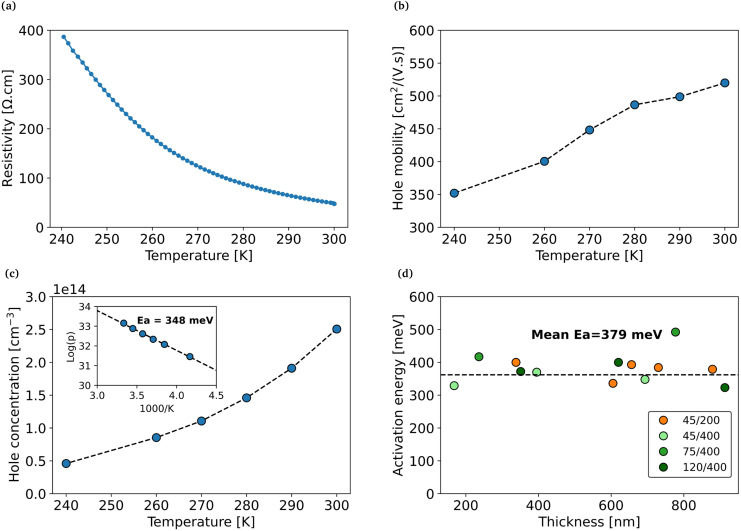
(a–c) Temperature-dependent Hall measurements for sample Z1. Temperature evolution of (a) resistivity, (c) mobility and (c) carrier concentration with corresponding fitting of the activation energy. (d) Activation energy for all measured samples.

Now that we have a better understanding of the microstructure, we will shift our focus to analyzing the electrical properties. Fig. 5 shows the temperature-dependent Hall measurement results for Z1, a sample of the 270 °C set grown from a 45/400 pattern. As the temperature decreases, the resistivity increases (Fig. 5a), which is expected for semiconductors. Hall measurements reveals also an appreciable increase in the carrier mobility (Fig. 5b). Based on this, we attribute the temperature dependence of the mobility to carrier scattering dominated by ionized defects, although unusual for a material with such a low carrier concentration in this temperature range. We speculate that this could be due to the presence of trap states or the presence of compensating donor defects. We would expect the latter to be ionized in this temperature range so that they could act as scattering centers while at the same time limiting the concentration of holes. The possible presence of intrinsic compensating donors is supported by a recent DFT study on Zn_3_P_2_.^8^ Additionally, misfit dislocations identified by HAADF STEM could also act as trap states.^35^

Finally, the activation energy can be extracted from the temperature evolution of the carrier density (Fig. 5c). All measured samples exhibit similar activation energies, ranging between 320–490 meV around a mean value of 379 meV (Fig. 5d). This value is much higher than the activation energies expected for zinc vacancies or phosphorus interstitials.^5,8,9^ This suggests that the rotated domains could play a role in the carrier concentration. In a recent study, Spadaro *et al.* showed that the interfaces between these domains contain dangling bonds which are associated with three energy levels located above the valence band: 100 meV, 210 meV and 380 meV.^30^ The deepest level matches well with the mean activation energy obtained in this work. The reason we see only this defect contribution and not the Zn vacancies could be explained by the temperature window of the measurement. Indeed, in this high-temperature range, a significant portion of the holes generated by the Zn vacancies are already excited to the valence band, while the holes that are located at the 380 meV level are only beginning to be excited.

### Effect of pattern on optoelectronic properties

**Fig. 6:**
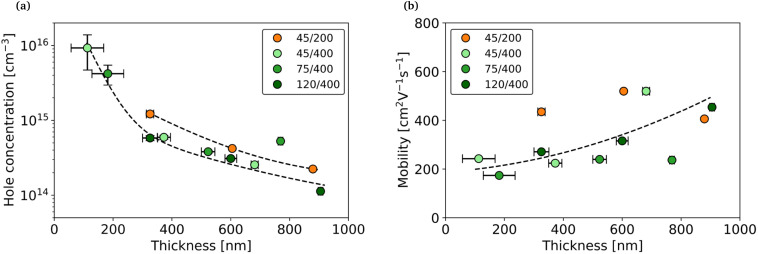
Evolution of (a) carrier concentration and (b) mobility with thickness for different patterns at room temperature. All samples are from the 270 °C set.

Temperature-dependent Hall measurements were performed on the set of samples grown at 270 °C. They all exhibit p-type conductivity, which is expected for undoped Zn_3_P_2_. Fig. 6a shows the evolution of carrier concentration with thickness at 300 K. The first observation is that for each pattern, the carrier concentration decreases with the thickness, going from approximately 10^16^ cm^−3^ to 10^14^ cm^−3^. Interestingly, we can observe a very clear trend for thin films with the same hole pitch. It seems that varying the hole size does not influence the carrier concentration. In contrast, thin films with smaller pitch exhibit a higher carrier concentration.

Fig. 6b shows the evolution of mobility with thickness for each pattern at 300 K. Again, the thin films with the same hole pitch follow roughly the same trend, with an increase in mobility with thickness. Similarly to the carrier concentration, we could explain this trend by a significant increase in the sensitivity to the defects at the interfaces for the thinnest films. This was already observed for other thin film semiconductors^36,37^ and more specifically for Zn_3_P_2_.^12^ For the samples with small pitch, the mobility is roughly constant with the thickness. The difference observed by varying pitch suggests that the dominant scattering mechanisms is surface scattering at the top interface. Indeed, a smaller pitch increases the density of lateral grain boundaries and the likelihood of rotate domains. At the same time - as already discussed - the top morphology becomes smoother with reducing pitch. Although one would reasonably expect a decrease in mobility with reducing pitch, our experimental observation points in the opposite direction, revealing the key role of top morphology. The highest mobility, reaching 563 cm^2^/V s, is obtained for a thin film of approximately 600 nm in thickness grown from a 45/200 pattern. It is the highest hole mobility ever measured for Zn_3_P_2_, consistently with the excellent crystalline properties. Moreover, the fact that there is no decrease in mobility for thicker films supports the hypothesis that we are not generating dislocations that would act as scattering centers.^12,38^

Our previous study on the electrical properties of Zn_3_P_2_ suggested that we could directly correlate the carrier concentration with the P/Zn ratio inside the material.^9^ However, in this work, the influence of the pattern on the carrier concentration is not consistent with the measured compositions. The samples with a 45/200 pattern have the highest carrier concentration even while having the lowest P/Zn ratio. Although we still believe that the generation of shallow acceptor defects related to an excess of phosphorus plays a role in the intrinsic doping of the material, it does not seem to be the dominating mechanism in thin films grown by selective area epitaxy. This may be due to differences between the two studies in the crystalline quality of Zn_3_P_2_ and in the types of defects presents. An other source of carriers could be the interfaces at the rotated domains. Although rotated domains in the film occur relatively randomly, we observed that they always appear at the Zn_3_P_2_/InP/SiO_2_ triple interface. These interfaces should be more prevalent for the 200 nm pitch samples, given that they possess four times more openings than the 400 nm pitch samples. However, it is unclear whether the total number of rotated domains interfaces is bigger for low pitch samples.

**Fig. 7:**
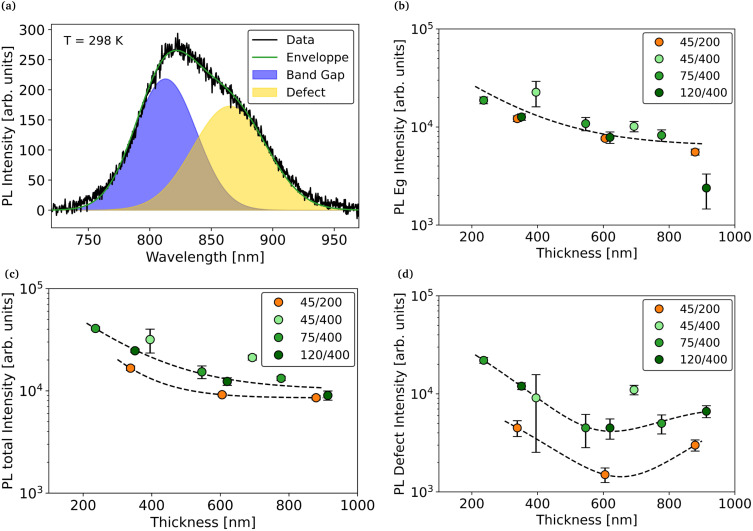
(a) Fitting of room temperature PL spectrum of sample Z2 with two emission peaks model. Evolution of the (b) total PL signal, (c) band gap PL signal and (d) acceptor defect PL signal with thickness for different patterns at room temperature. All samples are from the 270 °C set.

Photoluminescence measurements were performed to understand better the effect of the pattern on the optoelectronic properties of zinc phosphide. The spectrum of sample Z2, a thin film of the 270 °C set grown from a 120/400 pattern, is shown in Fig. 7a. It can be observed that there is not a single emission peak, but mainly two overlapping peaks. This is coherent to our previous studies, in which we showed that the PL spectrum of Zn_3_P_2_ is mainly composed of two main peaks.^4,39,40^ The first one, around 1.52 eV, is associated to the band gap of Zn_3_P_2_ (band-to-band emission) whereas the second one, around 1.4 eV, is associated to a defect band located slightly above the valence band.^40^ In previous studies, it was suggested that acceptor defects, such as phosphorus interstitial or zinc vacancies, could be at the origin of this band. Stutz *et al.* showed that a higher P/Zn ratio in the film induced a stronger emission of the defect peak.^4^ In our work, all the spectra were fitted in order to isolate both peaks. Furthermore, the Si_3_N_4_ protective layer on top induced Fabry–Perot interferences that need to be taken into account during the fitting.^41^ A comparison between samples with and without the Si_3_N_4_ layer, is shown in the SI.

Fig. 7b shows the evolution of the total PL signal with the thickness of the film for different patterns. We can see that the highest photoluminescence is obtained for the film with the smallest hole size and the largest pitch. The effect of the pitch on the luminescence is further supported by the comparison between samples of the 290 °C set with different pitches as shown in the SI.

Furthermore, for all patterns, the PL signal decreases with thickness. This trend is normally associated with the generation of trap defects during the growth, but this hypothesis is contradicted by the increase of mobility with thickness. Another explanation can be found in the evolution of the surface morphology with the thickness of the layer. As the growth continues, the texture of the surface decreases. As a result, the reflectivity of the surface increases. This causes both lower laser absorption and lower extraction of the emitted signal. To support this, the evolution of the PL intensity with the texturing, correlated with the simulated absorption is shown in the SI. Simulations were performed using the software OPAL 2.^42^

To better understand the effect of the pattern geometry on the total PL emission, we analyze the band gap (Fig. 7c) and the defect (Fig. 7d) peak intensities separately. One can see that the higher total PL intensity for the 45/400 samples is mostly due to a stronger band gap PL emission. We attribute the quenching of the band gap PL emission for larger hole size to the presence of the misfit dislocations at the Zn_3_P_2_/InP interface, which could act as non-radiative recombination centers.^43,44^ Similarly, the quenching of the band gap PL emission for smaller pitch could be due to the misfit dislocations located at the merging interface. Finally, Fig. 7d shows that the total PL quenching for the small pitch samples is mostly due to a weaker defect band emission. This is consistent with the lower P/Zn composition that was determined for the 200 nm pitch sample.

### Effect of growth conditions on optoelectronic properties

**Fig. 8:**
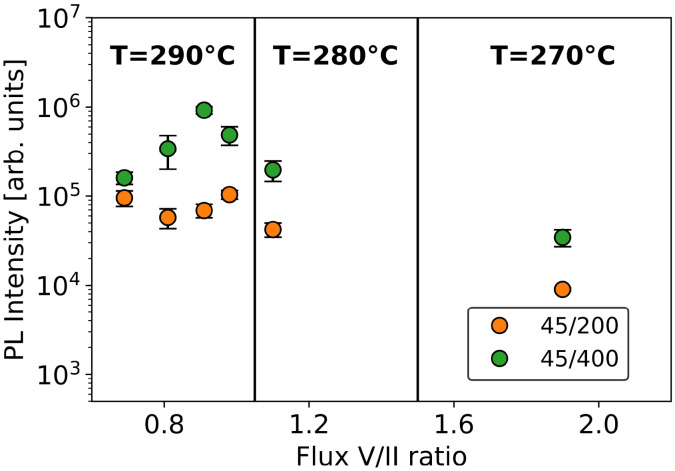
Evolution of the total PL signal with growth temperature and V/II ratio for two different patterns.

Room-temperature photoluminescence measurements were also performed on the *T*-variation set of samples. For this set, several samples were grown at 290 °C, varying the V/II ratio, and two additional samples were grown at lower temperatures. Fig. 8 shows the evolution of the total PL intensity with respect to the growth conditions for two patterns with different pitches. As observed before, we see that the PL intensity is always stronger for samples grown with the largest pitch. One should note that some 400 nm pitch samples have a notable strong PL emission. Those samples are thin and highly textured at the top, which reduces reflectivity and increases signal extraction. Furthermore, no clear trend emerges when only the V/II ratio is varied. Conversely, when the growth temperature decreases, there is a clear trend of decreased PL emission. However, we noticed that the 270 °C samples, despite having the lowest PL signal, actually have the highest hole mobility values as shown in the SI. The reason for these observations is quite unclear and should be investigated, which is outside the scope of this study.

## Conclusion

In conclusion, the high quality of Zn_3_P_2_ thin films grown by SAE was demonstrated and record values of electrical mobility shown. In particular, we correlate the improvement of the optoelectronic properties with the reduced presence of structural defects such as misfit dislocations (MD) and rotated domains (RD). We unveil the influence of the pattern on the occurrence of those defects and their impact on the optoelectronic properties of the thin films. We believe that the MDs, which were identified at the merging area for all samples and at the Zn_3_P_2_/InP interface for large hole size samples, act as non-radiative recombination centers which causes quenching of the PL intensity. On the other hand, our results suggest that the RD interfaces have an impact on the optoelectronic properties by causing an increase of the hole concentration and a quenching of the PL emission. Their predominant role in the conduction mechanisms is supported by the activation energies obtained from our measurement. Furthermore, the temperature evolution of the mobility suggests the presence of additional defects, acting as compensating donors. Also, the evolution of the electrical properties with the thickness indicates that either the top or the bottom interface contains a high density of defects correlated with an increase of the concentration of holes. Finally, we observed an increase of the PL emission when increasing the growth temperature. Hence, our results provide a correlation between the structural and the optoelectronic properties of SAE zinc phosphide and allows for a better understanding of the material with prospects of fabricating solar cells with improved efficiencies.

## Author contributions

The manuscript was written through contributions of all authors. RL and AFM conceived the experiments. RL performed the preparation of the substrate, the growth of the samples, the electrical characterization, the photoluminescence measurements and data analysis. RL and HRF performed FIB sample preparation. HRF performed as well the (S)TEM and EELS, and HRF, MCS and JA contributed to their data analysis. TH and DD contributed to the growth. LW conceived the idea for the hall measurements. CA contributed to the photoluminescence measurements. RL and HRF and wrote the original draft of the paper. All authors analyzed the corresponding data, discussed the results, and approved the final manuscript for submission.

## Conflicts of interest

The authors declare no conflicts of interest.

## Supplementary Material

TC-014-D5TC03582A-s001

## Data Availability

Raw data acquired at EPFL (Hall and PL measurements) are available at Science Data Bank at https://www.scidb.cn/en/s/zy63ym. Raw data acquired at ICN2 (STEM measurements) are available at CORA at https://doi.org/10.34810/data2688. Supplementary information (SI) is available. See DOI: https://doi.org/10.1039/d5tc03582a.
