# Supplementary material for: Nanopatterning optimization of zinc phosphide: hole mobility up to 560 cm2/V s with selective area epitaxy
Source: J Mater Chem C Mater. 2026 Apr 1;14(21):8950–9. doi: 10.1039/d5tc03582a (PMC13077530; doi:10.1039/d5tc03582a)
Supplement: TC-014-D5TC03582A-s001 [file TC-014-D5TC03582A-s001.pdf]

Supplementary Information (SI) for Journal of Materials Chemistry C.
This journal is © The Royal Society of Chemistry 2026

Supporting informations

Nanopatterning Optimization of Zinc Phosphide: Hole Mobility up to 560 cm^2/Vs with Selective Area Epitaxy

Raphael Lemerle,*‡[a] Helena R. Freitas,‡[b] Thomas Hagger,[a] Didem Dede,[a] Leo Webb,[a] Cynthia Aigroz,[a] Valerio Piazza,[a] Maria Chiara Spadarob,[b,c,d] Jordi Arbiol,*[b,e] and Anna Fontcuberta i Morral *[a,f]

[a] *Laboratory of Semiconductor Materials, Institute of Materials, School of Engineering, Ecole Polytechnique Fédérale de Lausanne, 1015, Lausanne, Switzerland.*

[b] *Catalan Institute of Nanoscience and Nanotechnology (ICN2), CSIC and BIST, Campus UAB, Bellaterra, Barcelona, Catalonia, 08193, Spain.*

[c] *Dipartimento di Fisica e Astronomia "Ettore Majorana", Università di Catania, via S. Sofia 64, Catania 95123, Italy*

[d] *IMM-CNR, Sede Catania Università, Via S. Sofia 64, 95123 Catania, Italy*

[e] *ICREA, Pg. Lluís Companys 23, 08010 Barcelona, Catalonia, Spain*

[f] *Institute of Physics, School of Basic Sciences, Ecole Polytechnique Fédérale de Lausanne, 1015, Lausanne, Switzerland.*

‡ These authors contributed equally to this work.

Substrate fabrication

Fe-InP (100) substrates (semi-insulating) were prepared by depositing 26 nm of silicon dioxide (SiO_2) using plasma-enhanced chemical vapour deposition (PECVD) in an Oxford Plasmalab System 100. The samples were then spin-coated with 40 nm ZEP resist before they were exposed at a dose of 200 uC/cm^2 using a Raith EBPG5000+ electron beam lithography system operating at 100 kV. The samples were then developed during 1 minute at room temperature using n-amylacetate. Then, the oxide was etched using SPTS APS Dielectic etcher for 26 seconds. The resist was then stripped by first rinsing the sample in acetone and IPA and second by exposing the sample under oxygen plasma for 7 minutes. Finally, a 10 seconds dip in a 1:39 BHF:H_2O solution was performed before introducing the substrates into the MBE system.

Device fabrication

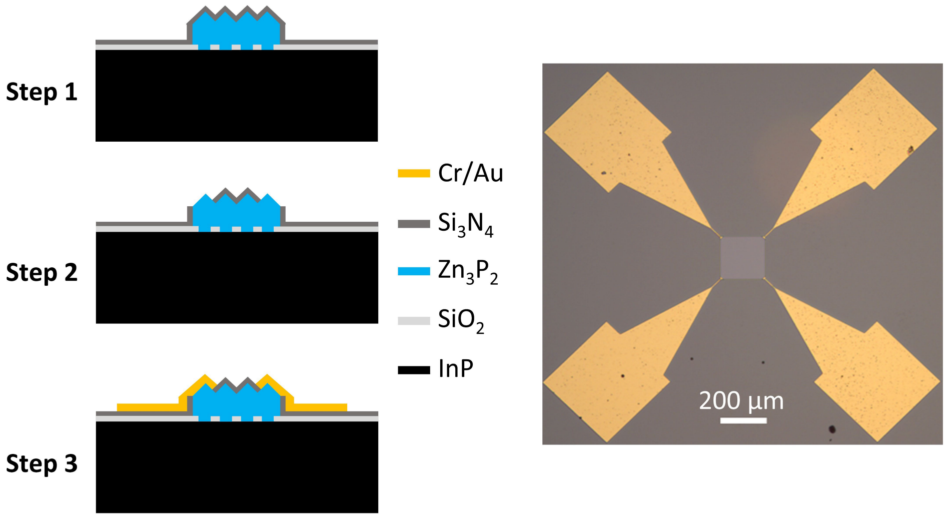

Fig. S1: Fabrication of the Van der pauw pattern on Zn_3P_2 thin films showing the process flow of the fabrications steps and an optical image of the device.

Hall measurement calculations - 3 configurations setup

The device is wire-bonded on a resistivity puck that can be then inserted into the PPMS. The puck has 3 bridge board channels, each with 4 contacts - one positive and one negative for current and voltage. This allows to perform up to 3 different measurements at the same time. Channel 2 is used to perform longitudinal measurement and extract the resistivity ρ, using the Van der Pauw equation:

$$\rho = \frac{\pi d}{ln(2)} \cdot R_2 \tag{1}$$

R_2 is the resistance measured in channel 2 and d is the thickness of the Zn_3P_2 thin film.

Channels 1 and 3 are used to perform transversal measurements under a magnetic field B and extract the carrier concentration. We use 2 channels in this case to check the symmetry of the measurements. From these measurements, we can get the hall coefficient R_H :

$$R_H = \frac{R_{1,3} \cdot d}{B} \tag{2}$$

From the hall coefficient, we can get the hole concentration p (with q the elementary charge) :

$$p = \frac{1}{R_H \cdot q} \tag{3}$$

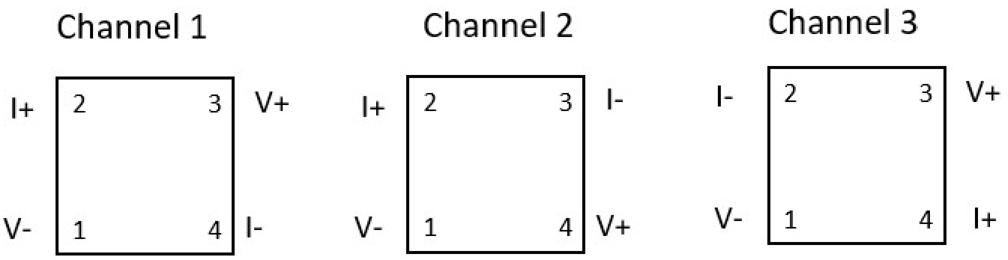

(a) Wiring configuration

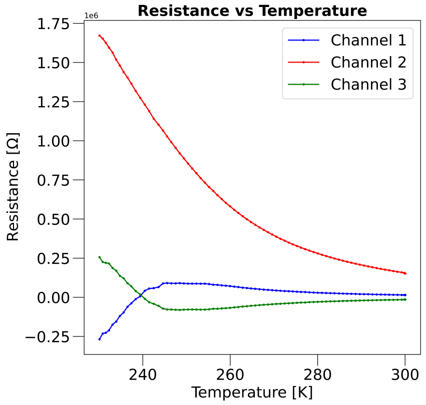

(b) Resistance with respect to temperature

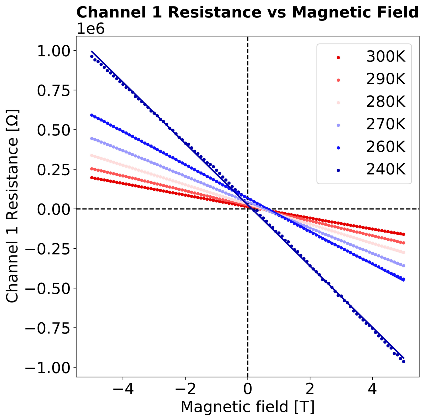

(c) Transversal resistance from ch1 with respect to magnetic field

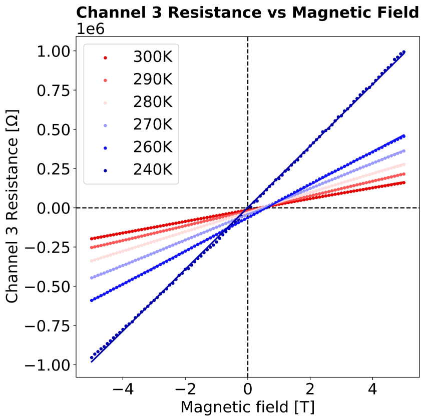

(d) Transversal resistance from ch3 with respect to magnetic field

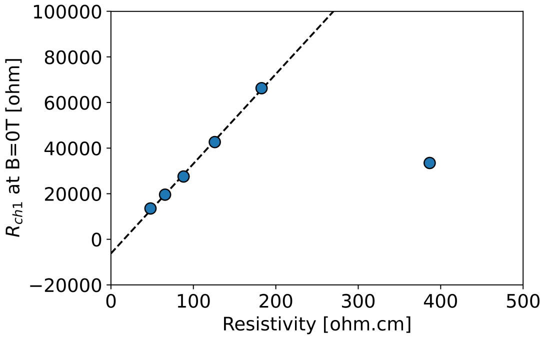

(e) Resistance offset for ch1 with respect to resistivity

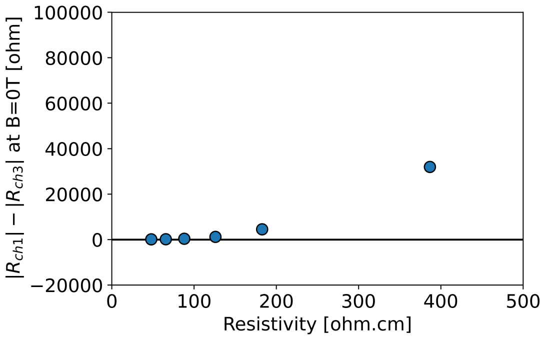

(f) Resistance offset difference between ch1 and ch2 with respect to resistivity

Fig. S2: Resistance measured from hall measurement for sample Z1. The negative slope for channel 1 and the positive slope for channel 3 indicate that the majority carriers are holes.

One way to verify the validity of the proposed two channel Hall effect technique is to examine the voltage offset with the magnetic field set to zero. We can then assess instrument and contact asymmetries and apply offset correction or nulling procedures to ensure reliable measurements. We know that the measured resistance includes not only the hall resistance but also a misalignment resistance R_m and a thermoelectric resistance R_{TE}[1]. The misalignment resistance is induced by any non-ideality in the geometry and is proportional to the resistivity ρ. The thermoelectric resistance is induced by any thermal gradients in the sample. In total, the measured resistance can be expressed by the following equation :

$$R_{measured} = \frac{R_H \cdot B}{d} + \alpha \cdot \frac{\rho}{d} + R_{TE} \quad (4)$$

The factor α describes the non-ideality of the geometry. At $B = 0$ T, the resistance offset should be proportional to the resistivity of the sample. As shown in Fig. S2.e, the resistance offset of channel 1 relative to the resistivity meets this criterion except at the lowest temperature. Fig. S2.f further shows that using both channels effectively suppresses the voltage offset, again with the lowest temperature measurement as the sole exception.

The calculation of the error bars for the carrier concentration is based on the error to determine the thickness d. Hall measurements equations were established for flat thin films. In our case, the samples have a significant surface roughness that increases when thickness decreases. The transport of charges in the pyramidal region is quite unclear, so we decided to set two limits for the effective thickness for carrier transport. The minimum limit is the thin film height (see Fig. S3), assuming no transport of charges in the pyramidal region. The maximum limit is the average between the thin film height and the pyramid height (see Fig. S3), assuming transport of charges everywhere in the pyramidal region.

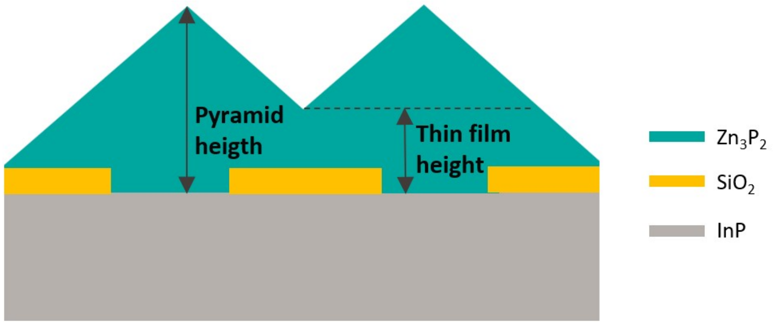

Fig. S3: Schematic of SAE Zn_3P_2 thin film on InP.

Hall measurement calculations - 12 configurations setup

In order to ascertain the veracity of the high value obtained for the mobility, we performed hall measurements using all the 12 configurations (8 longitudinal + 4 transversal) for the sample that exhibited the best mobility. The corresponding thin film has a thickness of 600 nm and is grown from a 45/200 pattern. The results are shown in table S1 and S2.

Table S1: Resistivity measured from 8 longitudinal configurations.

$V_{14}(I_{23})$	$V_{34}(I_{21})$	$V_{34}(I_{12})$	$V_{23}(I_{14})$	$V_{21}(I_{34})$	$V_{14}(I_{32})$	$V_{23}(I_{41})$	$V_{21}(I_{43})$	Mean	Stdev
40 Ω.cm	29 Ω.cm	26 Ω.cm	30 Ω.cm	26 Ω.cm	29 Ω.cm	30 Ω.cm	25 Ω.cm	29 Ω.cm	4.9 Ω.cm

Table S2: Carrier concentration measured from 4 transversal configurations.

$V_{31}(I_{24})$	$V_{31}(I_{42})$	$V_{24}(I_{13})$	$V_{24}(I_{31})$	Mean	Stdev
$3.5 \cdot 10^{14}$ cm^{-3}	$3.5 \cdot 10^{14}$ cm^{-3}	$4.1 \cdot 10^{14}$ cm^{-3}	$4.1 \cdot 10^{14}$ cm^{-3}	$3.8 \cdot 10^{14}$ cm^{-3}	$0.34 \cdot 10^{14}$ cm^{-3}

The mean mobility $< \mu >$ can be calculated from the mean resistivity $< \rho >$ and hole concentration $< p >$ with the following expression:

$$< \mu >= \frac{1}{q < \rho >< p >} \quad (5)$$

The standard deviation of the mobility σ_μ can be computed following the propagation of uncertainties :

$$\sigma_\mu = \sqrt{\left(\frac{\delta_\mu}{\delta_\rho}\delta_\rho\right)^2 + \left(\frac{\delta_\mu}{\delta_p}\delta_p\right)^2} \Rightarrow \sigma_\mu = \sqrt{\left(\frac{\sigma_\rho}{<\rho>}\right)^2 + \left(\frac{\sigma_p}{<p>}\right)^2} \quad (6)$$

We find that the mean mobility is equal to 563 $cm^2/(V.s)$ and the standard deviation to 107 $cm^2/(V.s)$.

Photoluminescence measurements

For every sample, the PL spectrum was fitted with two gaussians : one for the band gap emission peak and one for the acceptor defect emission peak. Furthermore, the Si_3N_4 layer on top induced Fabry–Perot interferences which need to be taken into account. The position and intensity of the peaks related these interferences changed with the top morphology of the sample. Fig. S4 shows how the partial removal of the Si_3N_4 layer by 1 min dipping in 1% HF influences those interference peaks. Finally, the error bars of the PL intensity was calculated by including the standard deviation of the 9 points of the map and fitting errors.

Fig. S5 shows the evolution of the total PL signal with the pyramid angle α of the films for different patterns. In addition, the simulated absorption of the laser in the film is also shown. The simulation is done using the OPAL 2 software. In the model used, we defined a layer of Zn_3P_2 on top of InP, with a pyramidal texturing. We set the optical constants of the films by using ellipsometry data aquired on Zn_3P_2 samples. A layer of 20 nm of Si_3N_4 on top of the film is also taken into account. We can see that the evolution of the PL intensity with the angle follows the same evolution than the simulated absorption, with an offset of roughly 10°. Finally, Fig. S6 shows the evolution of the total PL signal with the thickness of the films for different pitch.

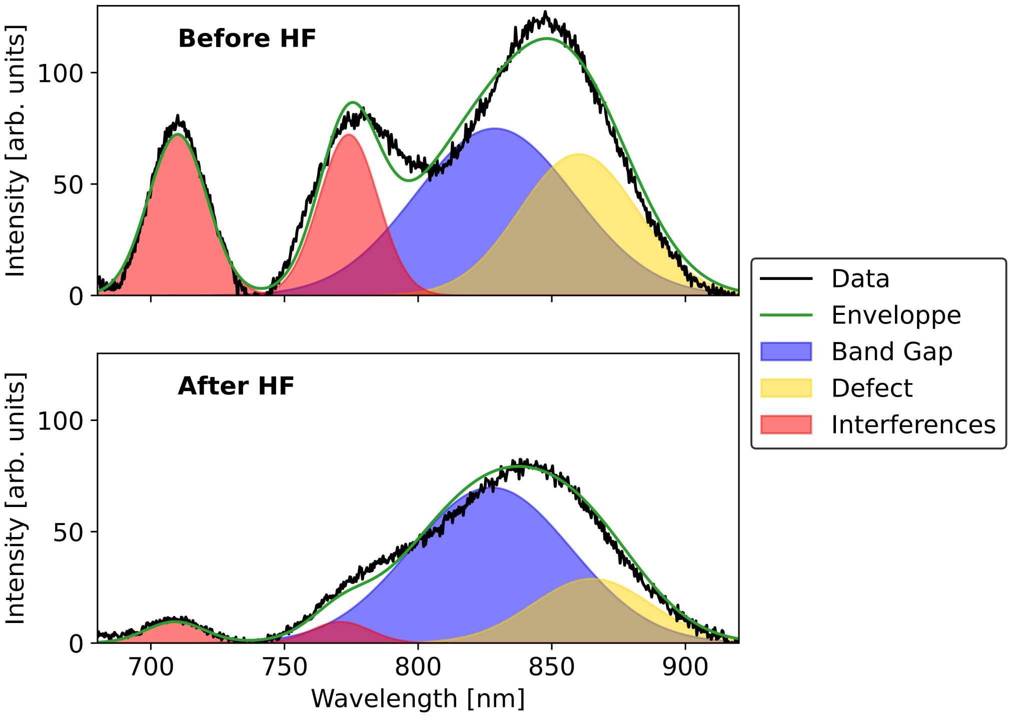

Fig. S4: Fitting of room temperature PL spectra, with model including interferences, of sample Z3 before and after 1 min in 1% HF.

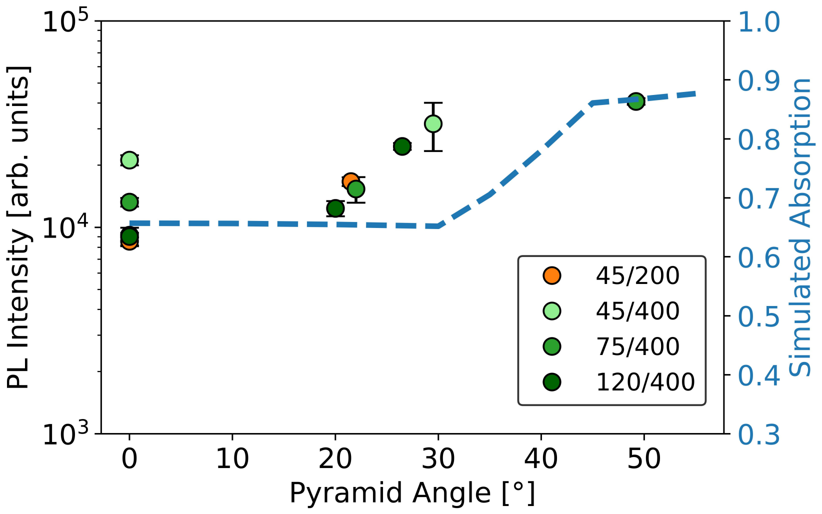

Fig. S5: Evolution of the total PL signal with angle α.

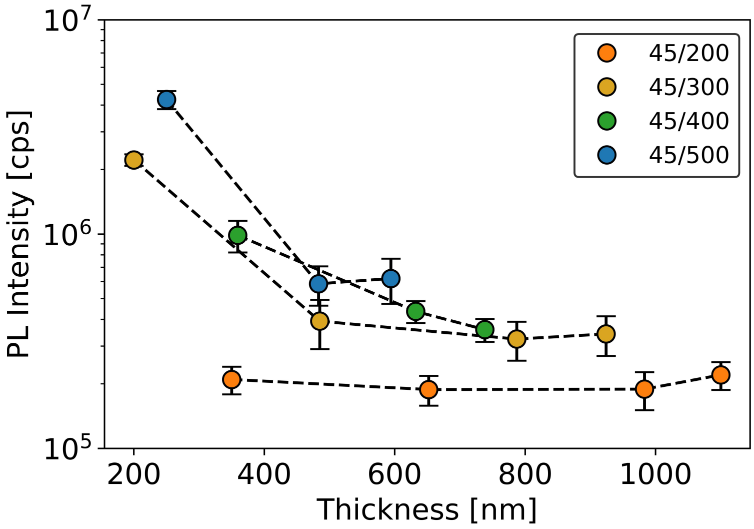

Fig. S6: Evolution of total PL signal with thickness for various pitch.

Hall measurements T-variation set

Hall measurements have been performed for samples of the T-variation set grown from a 45/200 pattern. Note that for one sample of this set, we could perform PL measurements but not hall measurements because of geometry constraints. Looking at the results, it is difficult to extract any clear trend for both carrier concentration and mobility.

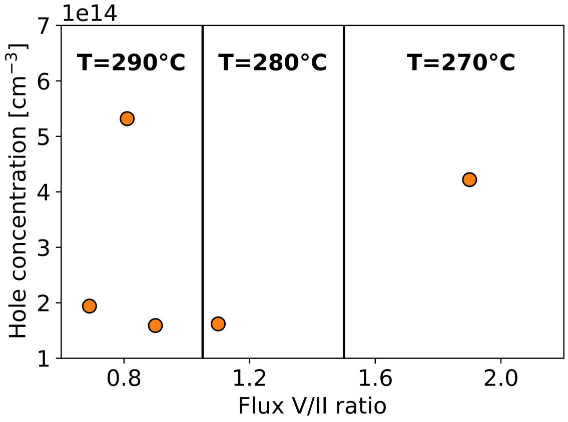

(a) Hole concentration

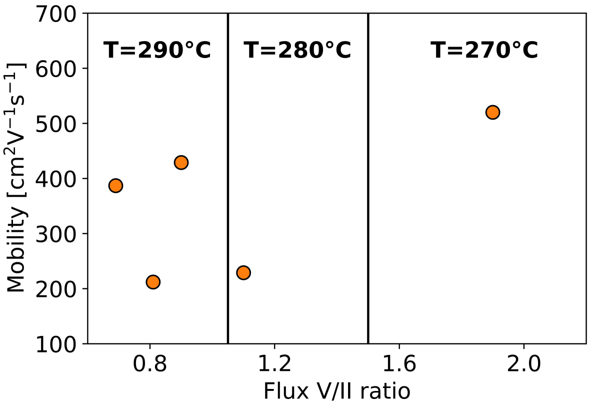

(b) Hole mobility

Fig. S7: Evolution of the electrical properties with growth temperature and V/II ratio for 45/200 samples.

References

[1] Jeffrey Lindemuth. *Hall Effect Measurement Handbook : A Fundamental Tool for Semiconductor Material Characterization.* Tech. rep. 2020.
